# Association between smoking and physical attractiveness: A longitudinal panel study from the China Family Panel Studies

**DOI:** 10.18332/tid/220847

**Published:** 2026-06-05

**Authors:** Jing Zhou, Qingsheng Liu, Fengrui Hua

**Affiliations:** 1 Research Institute of Social Development, Southwestern University of Finance and Economics, Chengdu, China; 2 School of Social Work and Health Management, Xihua University, Chengdu, China

**Keywords:** smoking, physical attractiveness, association, CFPS

## Abstract

**INTRODUCTION:**

Physical attractiveness constitutes a form of human capital, which shapes social interactions and life-course outcomes. This study investigated the association between smoking and physical attractiveness.

**METHODS:**

This longitudinal panel study conducted a secondary analysis of data from the China Family Panel Studies (CFPS) from 2010 to 2022, linking seven survey waves through unique individual and family identifiers. After restricting the analysis to respondents aged 16–85 years and applying additional data quality criteria, the final sample consisted of 33632 respondents and 119071 observations. The independent variables included smoking status and daily cigarette consumption. The dependent variable was the physical attractiveness evaluated by the interviewer, scored 1 to 7. To examine the association between smoking and physical attractiveness, we employed a two-way fixed-effects model that accounts for time-invariant individual characteristics and common time trends. Furthermore, we investigated the heterogeneity of this association with respect to gender, age, and marital status.

**RESULTS:**

A significant negative association existed between smoking and physical attractiveness in the analysis of 119071 observations. After adjusting for confounding variables, the average score for physical attractiveness of current smokers was 0.04 points lower than that of non-smokers (β= -0.04; 95% CI: -0.07 – -0.02). For every unit increase in the natural logarithm of daily cigarette consumption, physical attractiveness decreased by 0.02 points (β= -0.02; 95% CI: -0.03 – -0.01). Heterogeneity analyses indicated that this negative association was more pronounced among males, married individuals, and middle-aged and elderly adults (35–59 and ≥60 years), whereas it did not reach statistical significance among females, unmarried individuals, or young people (16–34 years).

**CONCLUSIONS:**

Smoking was negatively associated with physical attractiveness, which is more pronounced in certain populations. Further studies are needed to verify these findings and to explore whether appearance-based messaging could be used in tobacco control campaigns.

## INTRODUCTION

Smoking is a major risk factor for disease and mortality^1^. According to the Global Tobacco Epidemic Report 2025 from the World Health Organization (WHO), the global average smoking rate fell from 22.3% to 16.4% between 2007 and 2023, yet tobacco-related diseases still cause more than 7 million deaths each year^2^. Traditional anti-smoking campaigns emphasize risks such as lung, oral, gastric, and pancreatic cancers^3,4^. However, behavioral science suggests that the delayed and hidden nature of these risks reduces their effect on smokers’ short-term decisions^5^. Thus, negative outcomes that are more salient in perception and more outwardly observable may be worthy of consideration for public-health messaging.

Among the numerous negative outcomes associated with smoking, the change in physical attractiveness offers a promising entry point. This link rests on both physiological and psychological evidence. Physiologically, smoking damages skin collagen and elastic fibers, leading to deepened facial wrinkles, skin pigmentation, rough texture, and loss of elasticity^6-8^. Smoking contributes to dental plaque, tooth staining, and periodontitis. In addition to causing halitosis and hindering social interaction, it can also lead to gum recession and loose teeth, affecting appearance^9,10^. Psychologically, smoking as a visual cue can trigger negative trait inferences. On first impression, smokers are more likely to be perceived as lacking self-discipline, politeness, or honesty than non-smokers^11^. For instance, female smokers are more likely to be labelled as ‘rude’ or ‘slovenly’^12^, while fathers who smoke may face particularly harsh judgments for breaching family health responsibilities^13^. Consequently, the ‘appearance penalty’ of smoking encompasses both objective physiological changes and subjective social evaluations, constituting an immediate and socially significant burden.

Despite the increasing volume of relevant evidence, significant gaps remain. Most existing studies rely on small-scale clinical or cross-sectional designs, which lack longitudinal data from large populations. Furthermore, there has been limited examination of how this association may differ by age, gender, or marital status. To address these gaps, this study aims to investigate the association between smoking and physical attractiveness, as well as the potential variations across age, gender, and marital status.

## METHODS

### Data sources

This study uses data from the China Family Panel Studies (CFPS), a biennial survey conducted by the Social Science Survey Institute of Peking University from 2010 to 2022. The CFPS includes 25 provincial regions, using a stratified, multi-stage probability sampling design. Using unique household and individual identifiers to link the adult questionnaire data with household economic data across survey waves, we obtained 232711 original observations.

**Figure 1:**
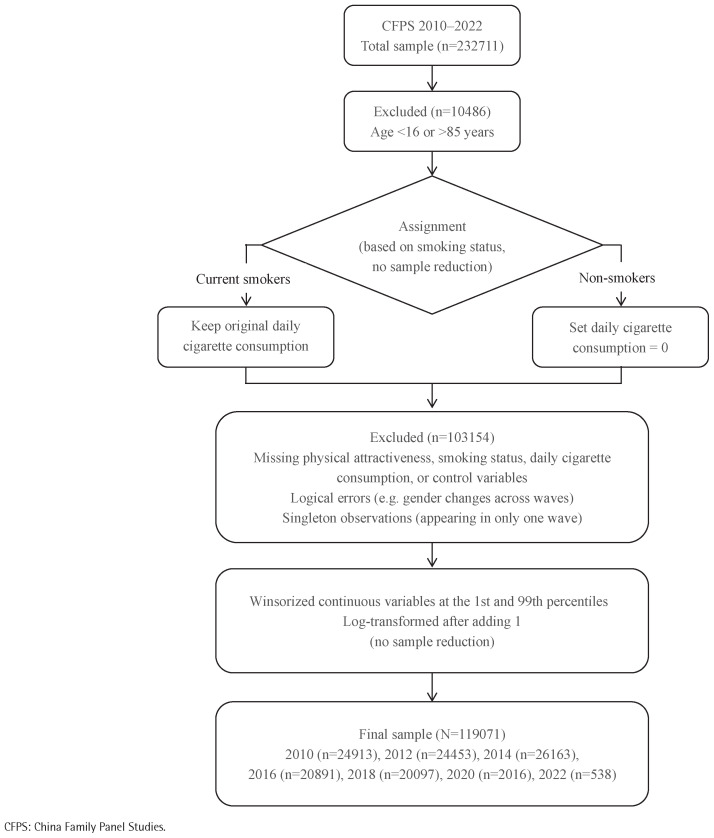
Participant screening flowchart, CFPS 2010–2022 (N=119071)

As shown in Figure 1, the sample screening process is as follows: 1) Following the definition of CFPS for adults, 10486 observations under the age of 16 or over the age of 85 years (the top 1% of the age distribution) were excluded; 2) In order to create a consistent measure, non-smokers were assigned a daily cigarette consumption of 0, while current smokers retained their original reported consumption. This step did not reduce the sample size; 3) A further 103154 observations were excluded due to missing physical attractiveness, smoking status, daily cigarette consumption, or control variables; logical errors such as gender changes across waves; or singleton observations appearing in only one wave. Missing data were handled by listwise deletion, and no imputation methods were used; 4) Continuous variables, including daily cigarette consumption and annual per capita household income, were winsorized at the 1st and 99th percentiles and then log transformed after adding 1. Body mass index (BMI) was only winsorized to avoid distortion of its interpretability through logarithmic transformation. This step also did not reduce the sample size.

After the above screening and processing, an unbalanced panel dataset comprising 33632 respondents and 119071 observations was obtained. Notably, the sample sizes for 2020 and 2022 were relatively small. During the COVID-19 pandemic, face-to-face interviews were replaced by telephone interviews, which resulted in substantial missing data on physical attractiveness^14^. Nevertheless, these two waves were retained to maintain the complete time span of the CFPS data.

### Variables

The independent variables were smoking status and daily cigarette consumption. Since 1991, the United States National Health Interview Survey (NHIS) has systematically used the ‘past 30 days’ as a time window to measure current smoking status, providing a standard method for identifying non-daily smokers in large-scale population surveys^15^. In accordance with this convention, smoking status is a binary variable derived from the CFPS questionnaire item: ‘Did you smoke in the past month?’ (yes, no). Daily cigarette consumption is a continuous variable based on the questionnaire item: ‘How many cigarettes do you smoke on average per day now?’.

The dependent variable was the interviewer’s rating of physical attractiveness, recorded in the final section of the CFPS adult questionnaire. Interviewers rated ‘The appearance of the respondent’ on a 7-point scale from ‘very poor’ to ‘very good’. This measure has good ecological validity and has been widely used in previous CFPS-based studies^16^. To mitigate potential bias, our two-way fixed-effects model includes individual fixed effects, which absorb time-invariant interviewer characteristics that might systematically affect their ratings.

Control variables were divided into individual- and family-level. All variables were considered potential confounders and included age, gender (female, male), marital status (single/divorced/widowed, married/cohabiting), household registration (non-agricultural, agricultural), education level (years), health status (measured on a 7-point scale, with higher scores indicating better health), BMI (kg/m^2^), alcohol consumption (<3, ≥3 times/month) and life satisfaction (on a 5-point scale from ‘very dissatisfied’ to ‘very satisfied’) . Control variables at the household-level included annual per capita household income in RMB (1000 Chinese Renminbi about US$150), and household size.

### Statistical analysis

All analyses were conducted using Stata/MP 18.0. Descriptive statistics were computed for all variables in the full sample and stratified by smoking status. Continuous variables are reported as mean ± standard deviation (SD) and were compared using independent-samples t-tests. Categorical variables are reported as frequency and percentage, and were compared using chi-squared tests. To examine the association between smoking and physical attractiveness, we employed two-way fixed-effects models with individual and year fixed effects. Although physical attractiveness is an ordinal measure (1–7), we used a linear fixed-effects model as our main specification. This approach accounts for time-invariant confounders and produces interpretable coefficients. We considered fixed-effects ordered logit models, but they require within-individual variation that was limited in our sample. Fixed-effects probit models are not available due to the incidental parameters problem. However, we also estimated a Poisson fixed-effects model, which is appropriate for ordered, count-like outcomes and efficiently accommodates high-dimensional fixed effects. All regressions used robust standard errors clustered at the individual level. Multicollinearity was assessed using variance inflation factors; the mean VIF was 1.31, and all individual VIFs were below 2, indicating no substantial multicollinearity. For heterogeneity analyses, we stratified the sample by gender, marital status, and age group (16–34, 35–59, and ≥60 years, based on China’s Medium- and Long-Term Youth Development Plan and the United Nations definition of older persons) and re-estimated the fixed-effects model in each subgroup^17,18^.

## RESULTS

### Characteristics of participants

Table 1 summarizes the descriptive statistics. The mean age of the sample was 47.35 years (SD=15.87), and 29.28% reported current smoking. Compared to non-smokers, current smokers were significantly older, predominantly male (OR=40.79; 95% CI: 38.84–42.84), and were more likely to be married (OR=1.46; 95% CI: 1.41–1.51). Regarding physical attractiveness, a lower proportion of current smokers received high scores (6–7 points) compared to non-smokers (45.76% vs 48.27%), while the share receiving low scores (1–2 points) was similar in both groups (1.62% vs 1.61%). Current smokers also had slightly higher education, higher BMI, and were more likely to consume alcohol, but had a slightly lower life satisfaction, lower household income, and smaller family sizes (p<0.001).

**Table 1:** Characteristics of participants by smoking status, CFPS 2010–2022 (N=119071)

*Variables*	*Overall* *(N=119071)* *n (%)*	*Non-smokers* *(N=84204)* *n (%)*	*Current smokers* *(N=34867)* *n (%)*	*p*
**Physical attractiveness** (1–7)				<0.001
1	378 (0.32)	266 (0.32)	112 (0.32)	
2	1540 (1.29)	1087 (1.29)	453 (1.30)	
3	7235 (6.08)	5028 (5.97)	2207 (6.33)	
4	19586 (16.45)	13613 (16.17)	5973 (17.13)	
5	33731 (28.33)	23563 (27.98)	10168 (29.16)	
6	34434 (28.92)	24490 (29.08)	9944 (28.52)	
7	22167 (18.62)	16157 (19.19)	6010 (17.24)	
**Age** (years), mean ± SD	47.35 ± 15.87	46.74 ± 16.38	48.83 ± 14.46	<0.001
**Gender**				<0.001
Female	60549 (50.85)	58688 (69.70)	1861 (5.34)	
Male	58522 (49.15)	25516 (30.30)	33006 (94.66)	
**Marital status**				<0.001
Single/divorced/widowed	20613 (17.31)	15833 (18.80)	4780 (13.71)	
Married/cohabiting	98458 (82.69)	68371 (81.20)	30087 (86.29)	
**Household registration**				<0.001
Non-agricultural	33515 (28.15)	24185 (28.72)	9330 (26.76)	
Agricultural	85556 (71.85)	60019 (71.28)	25537 (73.24)	
**Education level** (years), mean ± SD	7.19 ± 4.74	7.12 ± 4.89	7.35 ± 4.34	<0.001
**Health status** (1–7)				<0.001
1	696 (0.58)	533 (0.63)	163 (0.47)	
2	2062 (1.73)	1572 (1.87)	490 (1.41)	
3	6506 (5.46)	4839 (5.75)	1667 (4.78)	
4	16509 (13.86)	11846 (14.07)	4663 (13.37)	
5	31273 (26.26)	21937 (26.05)	9336 (26.78)	
6	36876 (30.97)	25660 (30.47)	11216 (32.17)	
7	25149 (21.12)	17817 (21.16)	7332 (21.03)	
**BMI** (kg/m^2^), mean ± SD	22.74 ± 3.36	22.71 ± 3.39	22.81 ± 3.30	<0.001
**Alcohol consumption** (times/month)				<0.001
<3	100056 (84.03)	77071 (91.53)	22985 (65.92)	
≥3	19015 (15.97)	7133 (8.47)	11882 (34.08)	
**Life satisfaction** (1–5)				<0.001
1	4317 (3.63)	2780 (3.30)	1537 (4.41)	
2	9244 (7.76)	6207 (7.37)	3037 (8.71)	
3	38661 (32.47)	26976 (32.04)	11685 (33.51)	
4	36390 (30.56)	26519 (31.49)	9871 (28.31)	
5	30459 (25.58)	21722 (25.80)	8737 (25.06)	
**Per capita income** (RMB)*, mean ± SD	9.08 ± 1.12	9.10 ± 1.12	9.04 ± 1.12	<0.001
**Household size**, mean ± SD	3.17 ± 1.36	3.18 ± 1.37	3.15 ± 1.35	<0.001

CFPS: China Family Panel Studies. p-values were derived from t-tests for continuous variables and chi-squared tests for categorical variables.

* Per capita income was logtransformed after adding 1. RMB: 1000 Chinese Renminbi about US$150.

### Association between smoking and physical attractiveness

Table 2 reports the benchmark regression results on the association between smoking and physical attractiveness. After controlling for individual and year fixed effects as well as other control variables, smoking was significantly negatively associated with physical attractiveness. The linear fixed-effects model indicated that, compared to non-smokers, current smokers had an average attractiveness score that was approximately 0.04 points lower (β= -0.04; 95% CI: -0.07 – -0.02). A one-unit increase in the natural logarithm of daily cigarette consumption was associated with a difference of -0.02 points in attractiveness, indicating that a 10% increase in daily cigarettes was associated with a 0.002 point lower attractiveness score (β= -0.02; 95% CI: -0.03 – -0.01). The Poisson fixed-effects model also showed a significant negative association for both smoking status (β= -0.01; 95% CI: -0.01 – -0.00) and daily cigarette consumption (β= -0.00; 95% CI: -0.01 – -0.00).

**Table 2:** Linear and Poisson regression results for the association between smoking and physical attractiveness, CFPS 2010–2022 (N=119071)

*Variables*	*Physical attractiveness (1-7)*			
	*Linear fixed-effect model*		*Poisson fixed-effects model*	
	*β (95% CI)*	*β (95% CI)*	*β (95% CI)*	*β (95% CI)*
**Smoking status**				
Non-smoker ®	0		0	
Current smoker	-0.04*** (-0.07 – -0.02)		-0.01*** (-0.01 – -0.00)	
**Daily cigarette consumption**		-0.02*** (-0.03 – -0.01)		-0.00*** (-0.01 – -0.00)
**Age** (years)	0.01 (-0.01–0.02)	0.01 (-0.01–0.02)	0.00 (-0.00–0.00)	0.00 (-0.00–0.00)
**Marital status**				
Single/divorced/widowed ®	0	0	0	0
Married/cohabiting	-0.03 (-0.06–0.00)	-0.03 (-0.06–0.00)	-0.01** (-0.01 – -0.00)	-0.01** (-0.01 – -0.00)
**Household registration**				
Non-agricultural ®	0	0	0	0
Agricultural	-0.01 (-0.04–0.02)	-0.01 (-0.04–0.02)	-0.00 (-0.01–0.01)	-0.00 (-0.01–0.01)
**Education level** (years)	-0.01** (-0.01 – -0.00)	-0.01** (-0.01 – -0.00)	-0.00*** (-0.00 – -0.00)	-0.00*** (-0.00 – -0.00)
**Health status** (1–7)	0.70*** (0.70–0.71)	0.70*** (0.70–0.71)	0.14*** (0.14–0.14)	0.14*** (0.14–0.14)
**BMI** (kg/m²)	-0.01*** (-0.01 – -0.00)	-0.01*** (-0.01 – -0.00)	-0.00*** (-0.00 – -0.00)	-0.00*** (-0.00 – -0.00)
**Alcohol consumption** (times/month)				
<3 ®	0	0	0	0
≥3	-0.03*** (-0.05 – -0.01)	-0.03*** (-0.05 – -0.01)	-0.01*** (-0.01 – -0.00)	-0.01*** (-0.01 – -0.00)
**Life satisfaction** (1–5)	0.01*** (0.00–0.01)	0.01*** (0.00–0.01)	0.00*** (0.00–0.00)	0.00*** (0.00–0.00)
**Per capita income** (RMB)	-0.00 (-0.01–0.00)	-0.00 (-0.01–0.00)	-0.00 (-0.00–0.00)	-0.00 (-0.00–0.00)
**Household size**	-0.00 (-0.01–0.00)	-0.00 (-0.01–0.00)	0.00 (-0.00–0.00)	0.00 (-0.00–0.00)
**Constant**	1.52*** (0.84–2.19)	1.51*** (0.84–2.19)	0.90*** (0.77–1.04)	0.90*** (0.77–1.04)
**N**	119071	119071	119071	119071
**R^2^**	0.74	0.74	-	-
**Individual FE**	Yes	Yes	Yes	Yes
**Year FE**	Yes	Yes	Yes	Yes

CFPS: China Family Panel Studies. FE: fixed effects. Daily cigarette consumption and per capita income were log-transformed after adding 1. The Poisson fixed-effects model does not report an R² value. Gender is absorbed by individual fixed effects. Coefficients rounded to two decimal places; 0.00 due to rounding. RMB: 1000 Chinese Renminbi about US$150. ® Reference category.

*** p<0.01,

** p<0.05, *p<0.1.

Among the control variables, health status exhibited a strong positive association with physical attractiveness in both linear fixed-effects models (β=0.70; 95% CI: 0.70–0.71). Life satisfaction was positively associated with attractiveness (β=0.01; 95% CI: 0.00–0.01), while alcohol consumption was negatively associated (β= -0.03; 95% CI: -0.05 – -0.01). Education level and BMI also demonstrated negative associations. The remaining control variables were not statistically significant.

### Subgroup analysis by gender, marital status and age

Table 3 presents the results of the subgroup analysis. Gender differences were pronounced. Among males, smoking was significantly associated with lower physical attractiveness (β= -0.05; 95% CI: -0.08 – -0.02), and physical attractiveness decreased with higher daily cigarette consumption (β= -0.02; 95% CI: -0.03 – -0.01). In contrast, the association was not significant in females. As for marital status, the significant negative association only existed in married groups, where both smoking status (β= -0.05; 95% CI: -0.08 – -0.03) and daily cigarette consumption (β= -0.02; 95% CI: -0.03 – -0.01) were significantly associated with reduced physical attractiveness. No significant association was found in unmarried groups. The most striking pattern, however, was age-related. Specifically, the effect was not significant in the young group in both models (16–34 years), while it emerged in the middle-aged group (35–59 years) (β= -0.05; 95% CI: -0.09 – -0.01; β= -0.02; 95% CI: -0.03 – -0.00). The strongest association was observed in the elderly group (≥60 years), with coefficients reaching their highest values (β= -0.09; 95% CI: -0.14 – -0.03; β= -0.04; 95% CI: -0.06 – -0.02).

**Table 3:** Subgroup analysis of the association between smoking and physical attractiveness by gender, marital status, and age, CFPS 2010–2022 (N=119071)

*Variables*	*Physical attractiveness (1-7)*						
	*Male*	*Female*	*Married/cohabiting*	*Single/divorced/* *widowed β (95% CI)*	*16–34 β (95% CI)*	*35–59 β (95% CI)*	*≥60*
	*β (95% CI)*	*β (95% CI)*	*β (95% CI)*				*β (95% CI)*
**Smoking status**							
Non-smoker ®	0	0	0	0	0	0	0
Current smoker	-0.05*** (-0.08 – -0.02)	-0.01 (-0.09–0.07)	-0.05*** (-0.08 – -0.03)	0.02 (-0.05–0.08)	-0.03 (-0.07–0.02)	-0.05*** (-0.09 – -0.01)	-0.09*** (-0.14 – -0.03)
N	58522	60549	96963	18880	26705	58427	25677
R^2^	0.73	0.75	0.73	0.79	0.77	0.73	0.73
**Daily cigarette consumption**	-0.02*** (-0.03 – -0.01)	-0.00 (-0.04–0.03)	-0.02*** (-0.03 – -0.01)	-0.00 (-0.03–0.03)	-0.01 (-0.04–0.01)	-0.02** (-0.03 – -0.00)	-0.04*** (-0.06 – -0.02)
N	58522	60549	96963	18880	26705	58427	25677
R^2^	0.73	0.75	0.73	0.79	0.77	0.73	0.73
**Control variables**	Yes	Yes	Yes	Yes	Yes	Yes	Yes
**Individual FE**	Yes	Yes	Yes	Yes	Yes	Yes	Yes
**Year FE**	Yes	Yes	Yes	Yes	Yes	Yes	Yes

CFPS: China Family Panel Studies. FE: fixed effects. Estimates from linear fixed-effects models. Daily cigarette consumption was log-transformed after adding 1. Control variables include age, gender, marital status, household registration, education (years), health status (1–7), body mass index (kg/m²), alcohol consumption, life satisfaction (1–5), log-transformed income (RMB) and household size. The stratifying variable is excluded in each subgroup model. Coefficients rounded to two decimal places; 0.00 due to rounding. Subgroup sample sizes do not sum to 119071 due to exclusion of subgroup-specific singleton observations. RMB: 1000 Chinese Renminbi about US$150. ® Reference category.

*** p<0.01,

** p<0.05, *p<0.1.

## DISCUSSION

Using national data from the CFPS, this study identified a significant negative association between smoking and physical attractiveness, particularly pronounced among males, married individuals, and middle-aged and elderly adults. These results echo and expand early experimental studies, including a twin study by Skinner et al.^19^ which found that smokers’ faces are perceived as less attractive^20^. This study further provides large-scale national evidence for the ‘appearance penalty’ of smoking, emphasizing its heterogeneity across different demographic groups.

The negative association between smoking and physical attractiveness may be attributed to physiological and psychosocial pathways. Physiologically, smoking has been shown to damage skin microcirculation, induce oxidative stress, destroy collagen and elastic fibers, and exert cumulative effects over time^21,22^. From a psychosocial perspective, smoking serves as an identity signal associated with negative personality traits, and the degree of stigmatization varies by social role^23-25^. Notably, interviewers need not have been aware of respondents’ smoking status; the visible consequences of smoking, such as wrinkles, skin discoloration, and tooth damage, may have unconsciously shaped their evaluations.

Subgroup analysis provides empirical evidence for how these two pathways operate across diverse populations. Among males, the appearance penalty was clear and pronounced, consistent with both mechanisms. Men’s skin is typically thicker and has more active sebum secretion, making smoking-induced roughness and dullness more likely to appear^26,27^. At the same time, there is a tension between smoking and the traditional male role expectation of maintaining a healthy appearance, which may induce stronger negative evaluations. The association among females did not reach statistical significance. The pattern by marital status is more sociopsychological in nature. The ‘appearance penalty’ for married current smokers was significantly larger than that for unmarried smokers, reflecting the convergence of dual pressures. Chronic stress associated with marital responsibilities may synergize with tobacco-related damage, accelerating the decline in physical attractiveness^28^. More importantly, marriage increases the visibility and normative scrutiny of health behaviors, thereby intensifying negative evaluations. In contrast, unmarried individuals tend to benefit from more diverse and dispersed social networks, which may reduce such stigma^29^. Age heterogeneity exhibits a clear age-gradient effect: during youth (16–34 years), when physiological functions remain vigorous, the microscopic damage from smoking may not yet be apparent^30^. With advancing age, especially after 35 years, repair capacity declines and cumulative harm becomes increasingly evident^31,32^. This damage acts synergistically with natural aging, producing the greatest decline in physical attractiveness in older adults (≥60 years)^33^. Social stigma toward smoking may also intensify in middle and later life, as the behavior increasingly conflicts with age-related expectations of maturity and responsibility. These findings are consistent with the view that smoking is associated with accelerated external aging^34^.

Given the modest effect sizes observed, we recognize the importance of distinguishing statistical from practical significance. Several considerations, however, suggest that they may still be worth attention from a public health perspective. First, the 7-point scale captures fine-grained distinctions; the same absolute difference would represent a larger proportion of the scale range if fewer categories were used. Second, smoking-related damage is likely cumulative. Although the effect is small at a single time point, repeated exposure may accumulate into a meaningful long-term decline. Furthermore, the modest overall effect size could be viewed alongside the substantial heterogeneity identified. The association was considerably stronger among males, married individuals, and older adults. Therefore, these findings may have cumulative relevance for public health messaging, particularly when targeted at higher-risk populations such as middle-aged men. For example, anti-smoking campaigns could use visual warnings that highlight premature aging signs in middle-aged male smokers or contrast skin health between young and older smokers.

### Limitations

This study has several limitations. First, causal relationships cannot easily be established. Although a two-way fixed-effects model is used to account for time-invariant confounders, some confounding variables (e.g. environmental exposure) could not be included due to data constraints. In addition to causal inference, self-reported smoking status may introduce information bias due to social desirability or recall bias. Interviewer ratings of physical attractiveness are inherently subjective and may introduce interviewer bias due to variations in aesthetic standards or cultural perceptions. Another limitation is related to statistical power. The proportion of female smokers in the sample is relatively small, limiting meaningful inference for this population. Finally, this study relied exclusively on data from the CFPS. The cultural and social context of China may limit the generalizability of our findings to other populations. Future research could integrate experimental designs for further validation and use standardized facial image acquisition (e.g. texture, symmetry, pigmentation) to reduce measurement bias. Cross-cultural studies using comparable data from other countries are also needed to test the cultural generalizability of these findings.

## CONCLUSIONS

Smoking is significantly negatively associated with physical attractiveness, and this negative association is more pronounced in certain demographic groups. Further research is needed to confirm these findings and to explore whether appearance-based messaging could be used in tobacco control campaigns for these high-risk populations.

## Data Availability

The data supporting this research are from the China Family Panel Studies (CFPS) and are available from the following link: https://cfpsdata.pku.edu.cn/#/home
